# Teaching the pediatric ear exam and diagnosis of Acute Otitis Media: a teaching and assessment model in three groups

**DOI:** 10.1186/s12909-017-0988-y

**Published:** 2017-08-29

**Authors:** Caroline R. Paul, Craig L. Gjerde, Gwen McIntosh, Lori S. Weber

**Affiliations:** 1 University of Wisconsin School of Medicine and Public Health, Madison, WI 53593 USA; 2 Department of Family Medicine and Community Health, 1100 Delaplaine Ct., Madison, WI 53715 USA; 3 Department of Pediatrics, Gundersen Health System, 1900 South Avenue, La Crosse, WI 54601 USA

**Keywords:** Clinical assessment, Pediatric Otoscopy, Acute Otitis media, Curriculum; learning about diagnosis

## Abstract

**Background:**

The serious consequences of inaccurate diagnosis of acute otitis media have led to a call for greater education to develop proficient pediatric otoscopy skills. Despite the clinical and educational needs, peer-reviewed standardized curricula with validated assessment instruments remain limited. This study evaluated a pediatric otoscopy curriculum incorporated into the Pediatric medical student clerkship with use of outcome measures that included assessment of skills with real patients. The objective was to determine whether students who received the intervention would demonstrate significant gains in pediatric otoscopy skills when compared with students with only routine immersion learning exposure.

**Methods:**

During their Pediatric clerkship, an intervention group (IG) of 100 medical students received routine instruction and a curriculum intervention. A non-intervention group (NIG) of 30 students received only routine instruction. Outcome measures included written tests and assessment of skills with real patients. A retention group (RG) consisted of 79 students in the IG who completed a written test at the end of medical school. Paired t-tests were used to compare differences in pre-intervention, post-intervention, and retention scores for the IG, NIG, and RG, while analysis of covariance tests were used to compare differences in scores between the IG and NIG.

**Results:**

Pre-intervention scores were similar for the IG and NIG for the written test (mean/SD of 12.9/2.9 for IG and 12.9/1.8 for NIG, *p* = 0.78) and skills checklist (mean/SD of 11.1/4.4 for IG and 10.9/4.0 for NIG, *p* = 0.88). The IG had significantly higher post-intervention scores than the NIG for the written test (mean/SD of 22.6/1.7 for IG and 13.9/2.5 for NIG, *p* < 0.001) and skills checklist (mean/SD of 19.2/3.4 for IG and 11.0/3.8 for NIG, *p* < 0.001). The IG also had significantly higher gain in scores than the NIG for the written test (mean/SD +9.6/2.8 for IG and +1.0/2.3 for NIG, *p* < 0.001) and skills checklist (mean/SD of +8.1/4.8 for IG and +0.1/4.5 for NIG, *p* < 0.001). For the RG, there was a significant decrease (*p* < 0.001) from the post-intervention scores to retention scores (mean/SD of −7.4/2.7) but a significant increase (*p* < 0.001) from the pre-intervention score to retention score (mean + 2.6/3.3).

**Conclusions:**

Medical students who received a formal curriculum intervention demonstrated significant gains in pediatric otoscopy skills when compared with students with only routine immersion learning exposure. However, learning gains diminished over time, emphasizing the need for continued practice opportunities to reinforce students’ skills. Our study provides a formal curriculum to meet identified educational gaps in the important topic of pediatric otoscopy and offers a model for teaching of other clinical skills using rigorous outcome measures including assessment of skills in real patients.

**Electronic supplementary material:**

The online version of this article (10.1186/s12909-017-0988-y) contains supplementary material, which is available to authorized users.

## Background

Acute otitis media (AOM) is the most frequently diagnosed illness in children. A recent prospective observational cohort study performed at multiple institutions in five European countries has documented an incidence of AOM of 256 cases per 1000 persons-years [1]. Furthermore, it has been shown that up to 75% of children will develop AOM at some time before the age of 5 years [2]. AOM is the most common indication for antimicrobial therapy [3, 4]. However, otitis media with effusion (OME), a condition often misdiagnosed as AOM and which does not require antibiotics, is actually the most common condition for which antibacterial agents are prescribed [5].

It is essential to correctly identify children with AOM, but the diagnosis is often challenging [4, 5]. While children with AOM typically present with clinical symptoms of fever, ear pain, and irritability, these findings are nonspecific and frequently overlap with OME and viral upper respiratory infection [6, 7]. Proficient skills in pediatric otoscopy is critical for making an accurate diagnosis of AOM as the condition is confirmed by the identification of an effusion and acute inflammatory changes in the middle ear. Diagnostic uncertainty due to a lack of pediatric otoscopy skills has led to an over-diagnosis of AOM, which has resulted in an increased incidence of antimicrobial resistance and higher healthcare costs due to unnecessary antibiotic prescriptions and surgical referrals [5].

The serious consequences of inaccurate diagnosis of AOM have led to a call for greater education regarding the diagnostic certainty of AOM [8]. Revised clinical guidelines from the American Academy of Pediatrics have specifically stressed that “educational and dissemination methods both at the practicing physician level and especially at the resident level need to be examined.” Furthermore, “instruction in the proper evaluation of the child’s middle ear status should begin with the first pediatric rotation in medical school and continue throughout postgraduate training” [5, 9].

Developing proficient skills in pediatric otoscopy is essential for the diagnosis of AOM [5, 9]. While formal curricula for pediatric otoscopy are emerging, studies have yet to assess the effectiveness of these interventions using real patients in a clinical setting [10–12]. Furthermore, to the best of our knowledge, curricula with formal standardized content and assessment instruments for medical students have not been described in the literature. While the topic of AOM is prevalent in the learning realm of the medical student, students report that their confidence at arriving at an accurate diagnosis of AOM is low [11]. To meet this educational gap with the overarching aim to impact patient care by improving the accuracy of diagnosing AOM, we evaluated a curriculum for pediatric otoscopy incorporated into the Pediatric medical student clerkship with the use of outcome measures that included assessment of skills with real patients. We hypothesize that students who received the formal curriculum intervention would demonstrate significant gains in pediatric otoscopy skills and the diagnosis of AOM when compared with students with only routine immersion learning exposure.

## Methods

### Study design

The objective of our study was to document significant gains in pediatric otoscopy skills and the diagnosis of AOM following a formal curriculum intervention. To meet this objective, medical students at the same university were divided into an intervention group (IG) performing their Pediatric clerkship at a large university hospital who received the curriculum and a non-intervention group (NIG) performing their Pediatric clerkship during the same time period at a large off-site community hospital who did not receive the curriculum. Gains in knowledge and skills over the course of the Pediatric clerkship were assessed using objective outcome measures. The study design allowed simultaneous enrollment of students at different institutions in the IG and NIG over the same time period to closely track them longitudinally, to enable outcome measures to be assessed in all students at the same time before and after the curriculum to decrease other factors that might influence post-intervention scores, and to prevent students in the NIG from becoming aware of the content of the curriculum and outcome assessment instruments through interactions with students in the IG.

### Subjects

The study was approved by our Institutional Review Board (IRB). The IG consisted of 100 consecutive third year medical students (47 males and 53 females with an average age 26.2 years) performing their Pediatric clerkship at a large university hospital who received the curriculum. A non-intervention group (NIG) consisted of 30 consecutive third year medical students (15 males and 15 females with an average age of 25.6 years) at the same university performing their Pediatric clerkship during the same time period at a large off-site community hospital with strong clinical, educational, and research affiliations with the university hospital. Training including the didactic lecture schedule, inpatient and outpatient clinical experiences, and educational objectives was similar for both groups except for the curriculum. Assignment of medical students to the university and community hospitals was based upon many factors including site availability and scheduling issues. A retention group was created to investigate whether gains in pediatric otoscopy skills and the diagnosis of AOM following the curriculum intervention could be maintained over time. The RG consisted of 79 students (38 males and 41 females with an average age of 25.8 years) in the IG who chose to complete a survey and written test at the end of medical school. Participation in the RG was voluntary as mandated by our IRB. Table 1 provides a comparison of students in the IG, NIG, and RG.

**Table 1:** Comparison of students in the intervention group (IG), non-intervention group (NIG), and retention group (RG)

	IG(*N* = 100)	NIG(*N* = 30)	RG(*N* = 79)	*P*-Value Between Groups
Proportion Males	47%	50%	48%	*P* = 0.84
Average Age (Years)	26.2(3.2 SD)	25.6(3.4 SD)	25.8(2.8 SD)	*P* = 0.54
Pre-Intervention Score on Written Test	12.9(2.9 SD)	12.9(1.8 SD)	13.0(2.3 SD)	*P* = 0.96
Pre-Intervention Score on Skills Checklist	11.1(4.4 SD)	10.9(4.0 SD)	11.2(4.2 SD)	*P* = 0.94

### Curriculum description

An IRB-approved needs assessment was performed on 88 consecutive third year medical students (43 males and 45 females with an average age of 25.9 years) during the prior year to determine learning needs and preferred learning modalities (Additional file 1). Based upon the needs assessment, a curriculum was developed as a two-hour “mini-lab” session performed in a clinical skills center which included a didactic lecture, a small group session focusing on clinical interpretation of tympanic membrane findings, and hands-on training. The curriculum’s content was adapted from Enhancing Proficiency in Otitis Media (ePROM), a peer reviewed web curriculum containing validated images and expert content [10]. A 12-item pediatric otoscopy skills checklist was developed which highlighted the key components of the approach to the pediatric ear exam and consisted of multiple content domains: discussion with the caregiver, equipment usage, child distraction techniques, holding positions, and specific portions of the exam including general technique, pneumatic otoscopy, and cerumen removal. The faculty used the skills checklist to demonstrate the proper method to perform the pediatric ear exam. Students used the checklist to practice otoscopy skills on each other and on mannequins chosen to represent children of varying ages and received facilitated faculty feedback on their skills in a serial manner until they demonstrated competent technique (Table 2).

**Table 2:** Description of the pediatric otoscopy curriculum

Curriculum Component	Description
Objectives	• Demonstrate Approach to Pediatric Ear Exam • Differentiate Clinically AOM, OME, and Normal Ear
Learning Methods	• Interactive Didactic Lecture • Small Group Discussion • Hands-On Training
Content	• Overview of Ear Anatomy • Discussion of Clinical Presentation and Diagnostic Criteria of AOM, OME, and Normal Ear • Illustration of Tympanic Membrane Findings Using ePROM Images • Discussion of Systematic Methods to Describe Tympanic Membrane • Live Demonstration of Pediatric Ear Exam by Faculty • Practice on Other Students and Mannequins Chosen to Represent Children of Varying Ages Using Skills Checklist with Facilitated Faculty Feedback
Outcome Measures	• Pre-Intervention and Post-Intervention Written Tests for Intervention Group and Non-Intervention Group • Pre-Intervention and Post-Intervention Assessment of Skills in Real Patients using Skills Checklist for Intervention Group and Non-Intervention Group • Post-Graduation Written Tests for Retention Group

### Outcome measures

The IG and NIG were evaluated with a written test and assessment of skills with real patients before and after the curriculum in order to determine whether students who received the intervention would demonstrate significant gains in pediatric otoscopy skills when compared with students with only routine immersion learning exposure. The written test consisted of 24 questions on description of the tympanic membrane and differentiation between AOM, OME, and the normal ear based upon images from ePROM [10]. The written test evaluated clinical skills in addition to actual knowledge as it contained validated images of the tympanic membrane and required the students to interpret the tympanic membrane findings. The pediatric otoscopy skills of the IG and NIG were also assessed by their clinical preceptor with real patients in the ambulatory care setting using the same 12-items skills checklist taught in the “mini-lab” session. The skills checklist assessed for both technical skills of the pediatric ear exam including equipment usage, holding positions, general technique, pneumatic otoscopy, and cerumen removal and behavior skills such as communication with caregivers and child distraction techniques [13]. The skills checklist underwent rigorous evaluation prior to its implementation including evidence of achieving feasibility, accuracy, validity, and inter-observer reliability [13].The IG and NIG received the pre-intervention and post-intervention written tests and assessment of skills with real patients at the same time in their Pediatric clerkship. At the end of medical school, the RG completed a survey regarding their learning experiences in pediatric otoscopy after their Pediatric clerkship and received a written test which was similar to the pre-intervention and post-intervention written tests (Table 2).

### Statistical analysis

Paired t-tests were used to compare pre-intervention and post-intervention scores for the IG and NIG for the written test and skills checklist. Analysis of covariance (ANCOVA) tests was used to compare pre-intervention, post-intervention, and gain in scores between the IG and NIG, with adjustment for potential co-variates of age and gender. Paired t-tests were used to compare pre-intervention, post-intervention, and retention scores on the written test for the RG.

## Results

For the written test, pre-intervention scores were similar (*p* = 0.78) for the IG and NIG, while post-intervention scores were significantly higher (*p* < 0.01) for the IG. There was a significant gain in scores for the IG (*p* < 0.01) and NIG (*p* = 0.03). The mean gain in scores was significantly higher (*p* < 0.01) for the IG than NIG (Table 3).

**Table 3:** Pre-intervention scores, post-intervention scores, and gain in scores on the written test and skills checklist for students in the intervention group (IG) and non-intervention group (NIG)

		Mean (SD)		*P*-ValueBetween Groups
		IG	NIG	
Written Test	Pre-InterventionScore	12.9 (2.9)	12.9 (1.8)	*P* = 0.78
	Post-InterventionScore	22.6 (1.7)	13.9 (2.5)	*P* < 0.001
	Gain in Score	+9.6^*^ (2.8)	+1.0^**^ (2.3)	*P* < 0.001
Skills Checklist	Pre-InterventionScore	11.1 (4.4)	10.9 (4.0)	*P* = 0.88
	Post-InterventionScore	19.2 (3.4)	11.0 (3.8)	*P* < 0.001
	Gain in Score	+8.1^*^ (4.8)	+0.1^***^ (4.5)	*P* < 0.001

^*^Statistically Significant Gain in Scores at *P* < 0.001

^**^Statistically Significant Gain in Scores at *P* = 0.03

^***^Non-Statistically Significant Gain in Scores at *P* = 0.90

For the skills checklist, pre-intervention scores were similar (*p* = 0.88) for the IG and NIG, while post-intervention scores were significantly higher (*p* < 0.001) for the IG. There was a significant gain in scores for the IG (*p* < 0.001) but not for the NIG (*p* = 0.90). The mean gain in scores was significantly higher (*p* < 0.001) for the IG than NIG (Table 3).

Sixty-eight percent of students in the RG reported no further gain of skills or practice opportunity in pediatric otoscopy after their Pediatric clerkship. There was a significant decrease (*p* < 0.001) in scores on the retention written test when compared to the post-intervention test but a significant increase (*p* < 0.001) in scores on the retention written test when compared to the pre-intervention test (Table 4).

**Table 4:** Pre-intervention scores, post-intervention scores, retention scores, and gain in scores on the written test for students in the retention group (RG)

		Mean (SD)	*P*-Valuev Between Scores
Pre-Intervention Score Versus Retention Score	Pre-Intervention Score	13.0 (2.3)	*P* < 0.001
	Retention Score	15.6 (2.5)	
	Gain in Score	+2.6^*^ (3.3)	N/A
Post-Intervention Score VersusRetention Score	Post-Intervention Score	23.0 (1.8)	*P* < 0.001
	Retention Score	15.6 (2.5)	
	Gain in Score	−7.4^**^ (2.7)	N/A

^*^Statistically Significant Gain in Score at *P* < 0.001

^**^Statistically Significant Regression in Score at *P* < 0.001

## Discussion

A gap in the literature exists regarding pediatric otoscopy and AOM despite these being important patient care topics and well-recognized educational needs [8]. Students currently learn these clinical topics through an informal immersion-type method with no measures to assess learning gains. We offer a formal curriculum intervention for teaching pediatric otoscopy and AOM and evaluating leaning gains which meets these educational needs. Our study demonstrated significant gains in knowledge and skills in pediatric otoscopy in medical students who received the curriculum when compared with students with only routine immersion learning exposure.

A crucial component of our curriculum was assessment of students’ pediatric otoscopy skills by their clinical preceptors with real patients. It is well recognized that curricula assessment instruments should measure gains in skills in direct patient care settings and that such gains should then translate into the ultimate outcome measure, namely improved patient outcomes [14, 15]. Yet, this translation of skills from the classroom to the bedside is typically assumed and rarely objectively evaluated. However, our study demonstrated that pediatric otoscopy skills gained in the curriculum intervention were translated into actual skills demonstrated with real patients which were objectively assessed using a validated skills checklist in a direct patient care setting.

Formal curricula for pediatric otoscopy are emerging but remain limited. Kaleida and associates first described the use of the ePROM web curriculum to teach pediatric otoscopy skills to residents but only used a written test as an outcome assessment measure [10]. Morris and associates reported the use of an ear simulator to improve the diagnostic accuracy of medical students to detect simulated ear effusions in mannequins [11]. Dinsmore and associates reported the use of a skills checklist to evaluate the otoscopy skills of audiology students using patient actors but did not describe a formal curriculum being evaluated [12]. There has been a recent emphasis in medical education to evaluate gains of clinical skills with real patients but not specifically with pediatric otoscopy [14]. To our knowledge, no previous studies have documented that pediatric otoscopy skills gained after a curriculum intervention could translate into actual skills demonstrated with real patients and assessed using a validated skills checklist.

However, our study also showed that significant learning gains could regress over time. This regression occurred not only with pediatric otoscopy, our selected topic here, but also with other skills which the clinical year student must learn and demonstrate proficiency including medical history taking [16], advanced cardiac life support [17], and airway intubation [18]. Previous studies have emphasized that continued re-assessment and practice opportunities are needed to reinforce students’ skills and diminish the risk of regression [16–18]. Unfortunately, our students at the end of medical school reported that they received few further real patient opportunities to practice their otoscopy skills.

Our study has limitations. While we compared gains in knowledge and skills between a NIG with only routine immersion learning exposure and an IG group that received a formal curriculum intervention, we acknowledge that our study was not a randomized control study or even a quasi-randomized study given the fact that the IG and NIG were of unequal sizes and performed their Pediatric clerkship at different hospitals. However, there was no significant difference in demographic factors and pre-intervention scores between the IG and NIG, and the clerkship grades, final written exam scores, and choice of residencies of medical students performing their Pediatric clerkship at the different hospitals have been closely tracked to ensure that all students receive the same educational experience. Nevertheless, we recognize that our study cannot account for differences in intelligence or prior pediatric otoscopy exposure between students, differences in clinical experiences provided by the university and community hospitals, or more immeasurable factors including “immersion effects”. Another limitation was that the retention of gains of the IG was only assessed using a written test as it was not possible to schedule time for students voluntarily participating in the RG to have their otoscopy skills assessed with real patients at the end of medical school. Finally, clinical preceptors could not be blinded to the groups being evaluated which may have created bias in their assessment of students’ otoscopy skills with real patients.

In conclusion, our study adds a formal curriculum intervention to the important topic of pediatric otoscopy which was evaluated using rigorous outcome measures and was found to yield significant gains including skills with real patients. The curriculum could be incorporated into the didactic lecture series included in the third year Pediatric medical student clerkship at most institutions and would provide an excellent complement to the routine immersion learning of pediatric otoscopy skills obtained in the hospital in-patient units and ambulatory clinics. However, learning gains diminished over time, emphasizing the need for continued practice opportunities to reinforce students’ skills. The educational content, multiple learning strategies, and rigorous assessment instruments described in our curriculum could be adapted for other clinical topics and other learner groups.

## Additional file

Additional file 1: Supplementary Files Need Assessment. (DOCX 13 kb)
