# Supplementary material for: Teaching the pediatric ear exam and diagnosis of Acute Otitis Media: a teaching and assessment model in three groups
Source: BMC Med Educ. 2017 Aug 29;17:146. doi: 10.1186/s12909-017-0988-y (PMC5574227; doi:10.1186/s12909-017-0988-y)
Supplement: Additional file 1: — Supplementary Files Need Assessment. (DOCX 13 kb) [file 12909_2017_988_MOESM1_ESM.docx]

APPENDIX 1

An IRB exempt needs assessment was performed for curriculum development purposes. Between July 2008 and June 2009 at the end of their Pediatric clerkship, an 18-item Likert-type survey was administered to 88 consecutive third year medical students (43 males and 45 females with an average age of 25.9 years). It focused on learning expectations and preferred learning modalities regarding pediatric otoscopy.

Response rate for the needs assessment used was 83% with 66 of the 88 students completing the survey. Ninety-seven percent of students had expected to learn how to perform the pediatric ear exam during their clerkship. Seventy-three percent of students reported anxiety when performing pediatric otoscopy, while 74% of students desired to have acquired more clinical skills. To increase their clinical skills, 89% of students preferred a “mini-lab” with hands-on training, 24% of students preferred lectures, and 44% of student’s preferred web-based learning. There was a significantly higher (p<0.05) proportion of students preferring the “mini-lab” with hands-on training to the other learning modalities.
